# The complexities of lithium

**DOI:** 10.14814/phy2.13405

**Published:** 2017-11-15

**Authors:** Benjamin Ko

**Affiliations:** ^1^ Section of Nephrology Department of Medicine University of Chicago Chicago Illinois

## Abstract

This is a editorial focus written to highlight the findings by Yang et al in the article “The Soluble (Pro)Renin Receptor Does Not Influence Lithium‐Induced Diabetes Insipidus but Does Provoke Beiging of White Adipose Tissue in Mice.”

Bipolar disorder is a common psychiatric condition characterized by periods of depression alternating with mania, affecting an estimated 1% of the population worldwide and 3% in the United States (Kessler et al. 2005). One of the mainstays of treatment for this disease is lithium, which is efficacious but has numerous side effects. Among these adverse effects is renal toxicity, including diabetes insipidus. The loss of urine concentrating ability in patients taking lithium is well documented, with some patients having urine outputs in excess of 10 L per day. Despite the widespread use of lithium, the mechanism by which lithium causes nephrogenic diabetes insipidus (NDI) remains incompletely characterized.

The downregulation of the water channel aquaporin 2 (AQP‐2) has long been thought be one of the causative factors in the pathogenesis of lithium‐induced NDI (Kwon et al. 2000). In prior work, the authors demonstrated that the soluble form of the (pro)renin receptor (sPRR) upregulated renal AQP‐2 expression (Lu et al. 2016). This resulted in an improvement in the urine concentrating ability in an experimental mouse model of NDI. In this issue of *Physiological Reports*, Yang et al., (2017) expand on this work, examining the role soluble pro‐renin receptor in the pathogenesis of lithium‐induced NDI. In the prior study, antagonism of the receptor for the AQP‐2 stimulatory hormone vasopressin (V2R) was used to generate mice with NDI. Here, to better study lithium‐induced NDI, the researchers induced NDI in a mouse model by administration of lithium chloride. This approach was successful in creating a state of NDI, resulting in a nearly tenfold increase in urine volume (9.86 ± 0.82 vs. 1.06 ± 0.09, *P* < 0.01) with corresponding changes in water intake (12.2 ± 0.78 vs. 3.06 ± 0.14, *P* < 0.01), urine osmolality (429.6 ± 29.3 vs. 2098.4 ± 170.2, *P* < 0.01) and plasma osmolality (310.0 ± 4.9 vs. 309.4 ± 3.5, *P* < 0.01). Western blotting demonstrated decreased abundance of AQP‐2 in lithium treated animals compared to control.

Coadministration of sPRR‐His, however, did not prevent the development of NDI. sPRR‐His combined with lithium chloride had no effect upon urine volume, water intake, urine osmolality or plasma osmolality compared to lithium chloride alone. Surprisingly, sPRR‐His had no change in expression of AQP‐2 at either the RNA or protein level.

The finding that sPRR‐His can induce AQP‐2 protein and activity in a V2R antagonism model but not in a lithium‐induced NDI model underscores the multifactorial nature of lithium‐induced NDI and the complex signaling pathways by which lithium acts. Multiple pathways are stimulated by lithium treatment (Lenox and Wang, 2003). Lithium is known to alter cyclic AMP, potentially decreasing AQP‐2 phosphorylation and activity, but this would be less likely to explain the lack of increased expression by sPRR (Li et al. 2006). The authors of this work also postulate roles for glycogen synthase 3B, purinergic signaling, and interstitial fibrosis in the pathogenesis of lithium‐induced NDI and note that the expression patterns of numerous proteins are altered by lithium. This study makes it abundantly clear that these pathways need to be investigated if we are to understand lithium‐induced NDI.

These findings clearly point the field of water channel research into exciting new directions and avenues of inquiry, but equally interesting, Yang et al. find an unexpected relationship between sPRR and adipose tissue. The researchers found a reduction in fat cell mass in sPRR‐His plus lithium‐treated mice at 14 days compared to lithium treated mice alone. Further examination of these animals revealed a “beiging” of the white adipose tissue with increased expression of UCP1, a marker of brown adipose tissue. The sPRR‐His group displayed lipid droplet morphology and mitochondrial content consistent with brown adipose tissue.

Adipose cells are characterized into white and brown adipose tissue (WAT and BAT, respectively). Cells with the characteristics of BAT appearing in WAT are often referred to “beige” adipose tissue. WAT and BAT perform many of the same functions but BAT contains a larger amount of mitochondria and are classically involved in thermogenesis, especially in the young. An understanding of the role of BAT in adults is growing, and now BAT is thought to play a role in weight loss and perhaps in preventing metabolic syndrome (Poekes et al. 2015).

Besides its renal side effects, lithium is well known to induce weight gain, with studies demonstrating that a 4 kg weight gain is average and a ten kilogram weight gain is typical in 20% (Vestergaard et al. 1988). Given the detrimental effects of obesity, a connection between sPRR and beiging of adipose tissue may have far‐reaching clinical applications. There are potential mechanistic links between sPRR and the regulation adipose tissue development. sPRR has been described to have effects on adipose tissue, with adipose‐specific deletion of sPRR associated with lipodystrophy (Wu et al. 2016). Furthermore, B‐catenin, a downstream mediator of sPRR, is a negative regulator of adipogenesis (Dogan et al. 2017). As with NDI, teasing apart the roles of these signaling pathways is needed to understand the beiging of adipose tissue. Importantly, is this effect of sPRR specific to co‐treatment with lithium or an independent effect?

In summary, this study by Yang et al. provides early mechanistic insight into two separate phenomena involving sPRR and lithium, showing that sPRR with lithium does not attenuate NDI but does result in the beiging of adipose tissue. Each of these findings evokes a number of questions that provide the basis for research in two separate fields, water channels and adipocyte biology, that will greatly further our understanding of the adverse effects of lithium.
